# Noise2Atom: unsupervised denoising for scanning transmission electron microscopy images

**DOI:** 10.1186/s42649-020-00041-8

**Published:** 2020-10-20

**Authors:** Feng Wang, Trond R. Henninen, Debora Keller, Rolf Erni

**Affiliations:** Electron Microscopy Center, Empa, Swiss Federal Laboratories for Materials Science and Technology, Überlandstr. 129, Dübendorf, CH-8600 Switzerland

**Keywords:** Denoising, STEM images, Deep learning, Unsupervised learning

## Abstract

We propose an effective deep learning model to denoise scanning transmission electron microscopy (STEM) image series, named Noise2Atom, to map images from a source domain $\mathcal {S}$ to a target domain $\mathcal {C}$, where $\mathcal {S}$ is for our noisy experimental dataset, and $\mathcal {C}$ is for the desired clear atomic images. Noise2Atom uses two external networks to apply additional constraints from the domain knowledge. This model requires no signal prior, no noise model estimation, and no paired training images. The only assumption is that the inputs are acquired with identical experimental configurations. To evaluate the restoration performance of our model, as it is impossible to obtain ground truth for our experimental dataset, we propose consecutive structural similarity (CSS) for image quality assessment, based on the fact that the structures remain much the same as the previous frame(s) within small scan intervals. We demonstrate the superiority of our model by providing evaluation in terms of CSS and visual quality on different experimental datasets.

## Introduction

Deep neural network denoising techniques have drawn a lot of attention(Kokkinos and Lefkimmiatis 2019; Zhang et al. 2018; Chang et al. 2019; Song et al. 2019; Lin et al. 2019; Lehtinen et al. 2018; Buchholz et al. 2019b; Zhang et al. 2019; Buchholz et al. 2019a; Guo et al. 2018; Kadimesetty et al. 2018; Liu et al. 2018; Mildenhall et al. 2018; Ran et al. 2019; Su et al. 2019; Xie et al. 2018) as they have significant impacts in addressing several drawbacks in conventional analytical methods (Lucas et al. 2018) such as (1) computation burden in the *testing* phase, i.e., an analytical method requires to resolve an optimization problem for every input, which is computationally inefficient, and (2) difficulties in setting up hyper-parameters to incorporate prior or domain knowledge. Deep Convolutional Neural Networks (DCNNs) are the default models of the choice when working with highly structured datasets such as images and videos, as DCNNs are (1) more computationally efficient than multilayer perceptron models featuring fewer parameters, and (2) take the advantages of the structured datasets such as translation invariance and locality.

While most of the DCNN models are trained using pairs of noisy and clean images, some of the recent methods, such as Noise2Void (Krull et al. 2019a), Noise2Self (Batson and Royer 2019) can be unsupervised, but at a price of degraded performance (Krull et al. 2019b). Noiser2Noise (Moran et al. 2020), probabilistic Noise2Void (Krull et al. 2019b) and parametric probabilistic Noise2Void (Prakash et al. 2020) (PPN2V) improve the performance by introducing estimated noise models.

With a typical dwell time of 10^−7^ s and down to less than 10 electrons per pixel, a modern scanning transmission electron microscopy (STEM) optimized for low-dose fast dynamic imaging produces very noisy images, often containing more noise than signal, as a result of high frames per second (fps) and the need to limit the radiation dose (Henninen et al. 2019). For training on such data, there do not exist any ground truth images. As modern electron microscopy experiments often target on studying complex dynamic systems of moving atoms (Cao et al. 2018; Henninen et al. 2019), it can be difficult to generate simulated images suitable as a ground truth. Therefore it is not feasible to denoise these images directly with supervised models. Furthermore, because of its inner complexity in data degradation, i.e., a simple additive white noise model does not comply (Wang et al. 2020), noise model based approaches are difficult.

Our approach assumes an underlying relationship between the clean atomic images and the noisy high-angle annular dark-field (HAADF) STEM images for our model to learn: for a bright area, there is a high possibility of the presence of atom(s), and for a dim area, there might only be the background. This relationship holds for our case when studying small metal atoms and clusters on lighter support films. Although we lack paired noisy-clean images, we can still train our model using Cycle-Consistent Adversarial Networks (Zhu et al. 2017). Moreover, we can apply an additional constraint to improve the restoration quality: a good model should give Gaussian-like shapes for atomic peaks(Dwyer et al. 2010). Our main contributions are:

1. demonstrating how to integrate domain-specific information with a Generative Adversarial Network (GAN) and a customized convolutional network extracting low-frequency features in a denoising application,
2. showing how to restore images using a cycle training strategy without knowing the signal prior and thus is free of noise models, and
3. proposing a quantitative metric for the image time series restoration where the ground truth does not exist.

## Methods

Our goal is to train a deep convolutional neural network translating the images in the domain $\mathcal {S}$ to another domain $\mathcal {C}$, where $\mathcal {S}$ is for the experimental noisy STEM images with training samples $\{s_{i}\}_{i}^{N} \in \mathcal {S}$, and $\mathcal {C}$ is for the expected atomic images composed of pure Gaussian peaks with simulated samples $\{c_{i}\}_{i}^{M} \in \mathcal {C}$. Our model includes two mappings $\mathbf {M}_{\text {s2c}}: \mathcal {S} \rightarrow \mathcal {C}$ and $\mathbf {M}_{\text {c2s}}: \mathcal {C} \rightarrow \mathcal {S}$. In addition, we introduce an adversarial discriminator **D** to distinguish between images {*c*} and translated images **M**_s2c_(*s*), an additional mapping **M**_*s*2*b*_ translating noisy images from domain $\mathcal {S}$ to domain $\mathcal {B}$, where $\mathcal {B}$ is for the slowly varying background of the noisy images, and a mapping $\mathbf {M}_{\text {s2b}}: \mathcal {S} \rightarrow \mathcal {B}$.

In our denoising application, we just need the model **M**_s2c_ to translate the noisy images from domain $\mathcal {S}$ to domain $\mathcal {C}$. As there are no paired training images available, we cannot train this model in an end-to-end fashion. To make the training feasible, we employ the cycle training scheme by introducing a second model **M**_c2s_. With this additional model, we can train model **M**_s2c_ by training

1. the composed model **M**_s2c_∘**M**_c2s_ by mapping noisy images to noisy images, and
2. the composed model **M**_c2s_∘**M**_s2c_ by mapping clear images to clear images.

The objective of our model contains three terms:

1. *an adversarial loss* for matching the distributions of the denoised images to the data distributions of the simulated atomic images,
2. *a cycle consistency loss* to prevent the learned composed mapping **M**_s2c_∘**M**_c2s_ from contradicting to the original, which is inspired by CycleGan (Zhu et al. 2017), Dualgan (Yi et al. 2017) and DiscoGan (Kim et al. 2017), and
3. *a low-frequency cycle loss* to keep the learned composed mapping **M**_s2b_∘**M**_c2s_∘**M**_s2c_ staying consistent with the low-frequency components.

### Adversarial loss

We apply adversarial loss to mapping **M**_s2c_. For this mapping and its adversarial **D**, with a batch of training samples $\mathbf {s} \in \mathcal {S}$ and $\mathbf {c} \in \mathcal {C}$, the objective contains two adversarial losses and a gradient penalty loss

1 $$ \mathcal{L}_{\text{gan}} = W(-1, \mathbf{D}(\mathbf{s})) + W(1, \mathbf{D}(\mathbf{M}_{\text{s2c}}(\mathbf{s})) + \lambda * G(\mathbf{s}),  $$

in which *W* is the Wasserstein loss function(Arjovsky et al. 2017; Wu et al. 2018), and in our implementation

2 $$ W(x, y) = \mathbb{E}[x \odot y],  $$

where *G* is the gradient penalty loss function, for a single sample *s*_*i*_∈**s**, with the prediction of *x*_*i*_=**M**_s2c_(*s*_*i*_) and the random weighted sample *y*_*i*_=*R*(*s*_*i*_,*x*_*i*_). The penalty loss for these samples is

3 $$ G'(s_{i}) = {\|} 1 - \sqrt{\sum ({\partial{x_{i}}}/{\partial{y_{i}}})^{2}} {\|}^{2}  $$

in which *R* is the randomized weighting function with a uniform random tensor *u* in range [0,1]

4 $$ R(x, y) = u \odot x + (1-u) \odot y,  $$

and *G* is an average over the training batch size *b*_*s*_

5 $$ G(\mathbf{s}) = \frac{\sum^{b_{s}}_{i=1} G'(s_{i})}{b_{s}},  $$

*λ*=10 controls the gradient penalty strength, ⊙ denotes elementwise multiplication and $\mathbb {E}$ denotes the mean of the elements.

### Cycle consistency loss

We expect the clean images translated to domain $\mathcal {S}$ with **M**_c2s_ could be translated back to the domain $\mathcal {C}$ with **M**_s2c_, without changing any of the contents. To apply this constraint, we use the cycle consistency loss

6 $$ \mathcal{L}_{\text{cycle}} = \mathbb{E}\|\mathbf{c} - \mathbf{M}_{\text{s2c}}(\mathbf{M}_{\text{c2s}}(\mathbf{c}))\|.  $$

### Low-Frequency cycle loss

We realize that the noises in the experimental images are typically random discrete bright and dark pixels, therefore we relax **M**_*c*2*s*_ by comparing the low-frequency features of its outputs with the inputs, instead of enforcing exactly the pixel-wise matching. We express this objective as

7 $$ \mathcal{L}_{\text{lfc}} = \mathbb{E}\|\mathbf{M}_{\text{s2b}}(\mathbf{s}) - \mathbf{M}_{\text{s2b}}(\mathbf{M}_{\text{c2s}}(\mathbf{M}_{\text{s2c}}(\mathbf{s})))\|,  $$

in which **M**_s2b_ is manually designed with precalculated Gaussian filters.

### Full objective

The full objective is

8 $$ \mathcal{L}_{n2a} = \alpha \mathcal{L}_{\text{gan}} + \beta \mathcal{L}_{\text{cycle}} + \mathcal{L}_{\text{lfc}},  $$

in which the constants *α*=5 and *β*=1 control the relative weights of the three losses. Finally, we aim to solve

9 $$ \mathbf{M}_{\text{s2c}}^{*} = {\underset{\mathbf{M}_{c2s},\mathbf{M}_{s2c}}{\arg \min}} \underset{\mathbf{D}}{\text{max}} \mathcal{L}_{\text{n2a}}.  $$

### Implementation details

**Datasets.** There are two types of images:

1. *Simulated clean atomic images:* the images in domain $\mathcal {C}$. We simulated 32768 clean images. First, we randomly sampled 75–150 atomic positions in a 2D 256×256 pixel lattice. Then, we randomly assigned 1–4 atoms to each of the positions. Afterward, we generated a 33×33 pixel 2D Gaussian kernel with a random variance in the range [1.0,10.0]. Then, this kernel was convolved with the 2D lattice, and the 128×128 pixels from the center of the image was cropped as our simulated clean image.
2. *Experimental STEM images*: the images in domain $\mathcal {S}$. We tested our approach on three experimental movies of dynamic atomic clusters of Pt on a carbon film (Henninen et al. 2019, 2020). This data was recorded at 150 fps with 128×128 pixels, 15 fps with 512×512 pixels and 5 fps with 1024×1024 pixels, with an electron dose in the range [10^5^,10^6^]*e*Å ^−2^*s*^−1^ using a FEI Titan Themis, operated at 300 kV.

**Network Architectures.** We design **M**_s2c_ and **M**_c2s_ as two identical U-Nets (Ronneberger et al. 2015) of depth 3 with Xception modules (Chollet 2017) using kernel sizes {1,3,5,7}, deep residual blocks (He et al. 2016) of 16, instance normalization (Ulyanov et al. 2016) followed by leaky relu activation, except a tanh activation function at the last layer. For upsampling, we use a transposed convolution with a stride of 2 and a kernel size of 4×4, followed by a convolution with a kernel size of 3×3. For downsampling, we use transpose convolution with a kernel size of 3×3, followed by a convolution with a stride of 2 and a kernel size of 3×3. There are no zero-paddings applied to the convolution and transpose convolution operations. These two models aim at translating noisy images to clean images and at translating clean images to noisy images, respectively. We design **M**_s2b_ as a one-layered network, using a single filter of size 33×33, without padding and bias. Moreover, we precalculate the weights as a normalized 2D Gaussian distribution with a variance of *σ*=15.0. This model aims to match the slowing varying low-frequency features of two noisy images. The critic model **D** is composed of 4 downsampling modules and a fully connected layer. The downsampling modules contain a transposed convolution layer with a kernel size of 3×3, followed by a convolution layer of stride 2 with a kernel size of 3×3 and then a dropout layer of 25%. This model aims to classify whether an input image only contains 2D Gaussian-like peaks or not.

**Fig. 1:**
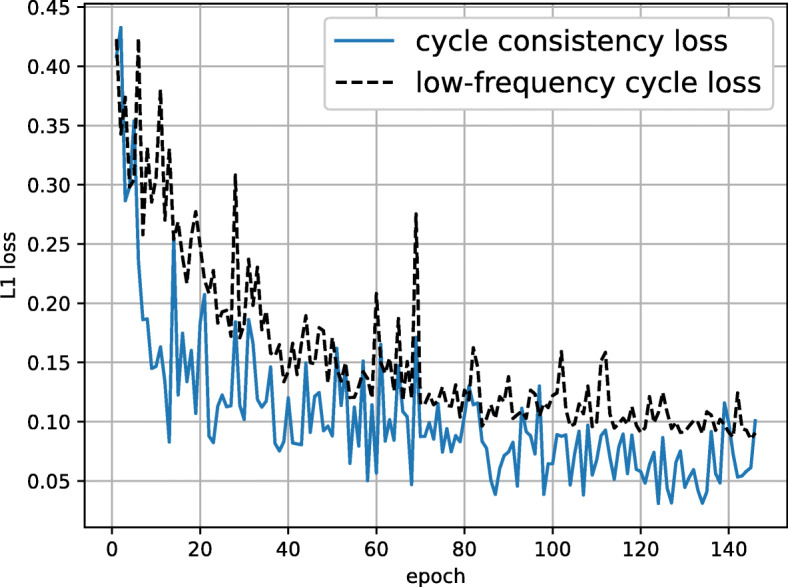
Typical loss curves for $\mathcal {L}_{\text {cycle}}$ and $\mathcal {L}_{\text {lfc}}$

**Training Details.** Our model contains hundreds of layers, to fit all the data into the 12 GB memory of an Nvidia GTX 1080 Ti GPU, we crop our experimental images into 128×128 pixels, and train using RMSProp algorithm (Krizhevsky et al. 2012) with a batch size of 6, a learning rate of 5×10^−5^ and a momentum of 0.9. A typical aberration-corrected HAADF STEM dataset could contain thousands of images. To save the computation time, for each dataset, 120 images are randomly sampled to train our model. Our model usually takes 100 epochs to reach a good prediction, when the cycle consistency loss is less than 0.05 and the low-frequency cycle loss is less than 0.1. Typical convergence curves for $\mathcal {L}_{\text {cycle}}$ and $\mathcal {L}_{\text {lfc}}$ are presented in Fig. 1.

**Fig. 2:**
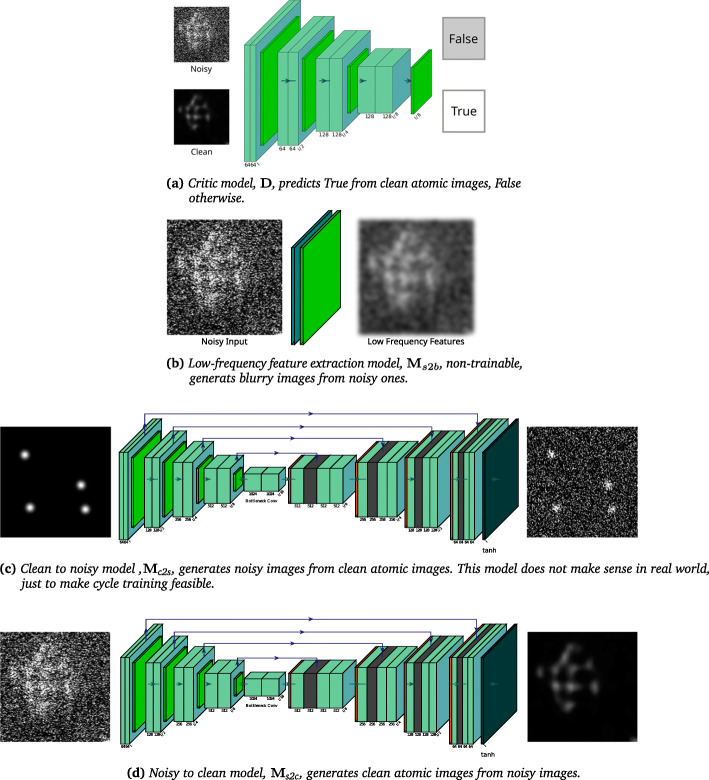
Submodels in Noise2Atom
② a noisy to clean model, **M**_s2c_, that translates noisy images to clean images, as is demonstrated in Fig. 2d,
③ a clean to noisy model, **M**_c2s_, that translates clean images to noisy images, as is demonstrated in Fig. 2c, and
④ a low-frequency feature extraction model, **M**_*s*2*b*_, that translates noisy images to blurry images, as is demonstrated in Fig. 2b.

Explicitly, Noise2Atom is composed of 4 sub-models:

① a critic model, **D**, that predicts *True* on clean images and *False* on noisy images, as is demonstrated in Fig. 2a,

With the weights for sub-model ➃ already hand-crafted, we train sub-models ➀ – ➂ in this fashion (in a single batch):

1. training sub-model ➀ **5 times** by mapping noisy images to *False* and clean images to *True*,
2. training composed model ➁ ∘➀ **once** by mapping noisy images to *True*, with sub-model ➀ fixed,
3. training composed model ➁ ∘➂ ∘➃ **once** by mapping noisy images to their low-frequency features, with sub-model ➃ fixed, and
4. training composed model ➂ ∘➁ **once** by mapping clean images to themselves.

## Results

There do not exist any noise-free images as ground truth for our experimental datasets. Therefore, for evaluating the restoration quality, we design a consecutive similarity (CSS) metric. For this, we assume most of the contents (except for noise) of neighboring consecutive frames remain the same from frame to frame, due to the short frame time (typically 10^−1^ s). The CSS metric for the image *I*_*n*_ at frame *n* and the image *I*_*n*+1_ at frame *n*+1 is given by

10 $$ {}CSS(I_{n}, I_{n+1}) = \frac{(2\mathbb{E}_{I_{n}}\mathbb{E}_{I_{n+1}}+C_{1})(2\sigma_{I_{n},I_{n+1}}+C_{2})}{\left(\mathbb{E}_{I_{n}}^{2}+\mathbb{E}_{I_{n+1}}^{2}+C_{1}\right)\left(\sigma_{I_{n}}^{2}+\sigma_{I_{n+1}}^{2}+C_{2}\right)},  $$

in which $\sigma _{I_{n}}^{2}$ and $\sigma _{I_{n+1}}^{2}$ are the variance, $\sigma _{I_{n},I_{n+1}}$ is the covariance of *I*_*n*_ and *I*_*n*+1_,*C*_1_=10^−4^ and *C*_2_=9×10^−4^ are two constants to stabilize the division. The CSS metric is a variation of the structural similarity index measure (SSIM), which is widely used to predict the reconstruction quality by measuring the similarity between the ground truth image and the predicted image. As we do not have ground truth in this domain specific problem, we predict the denoising quality by measuring the similarity between two denoised consecutive images.

**Fig. 3:**
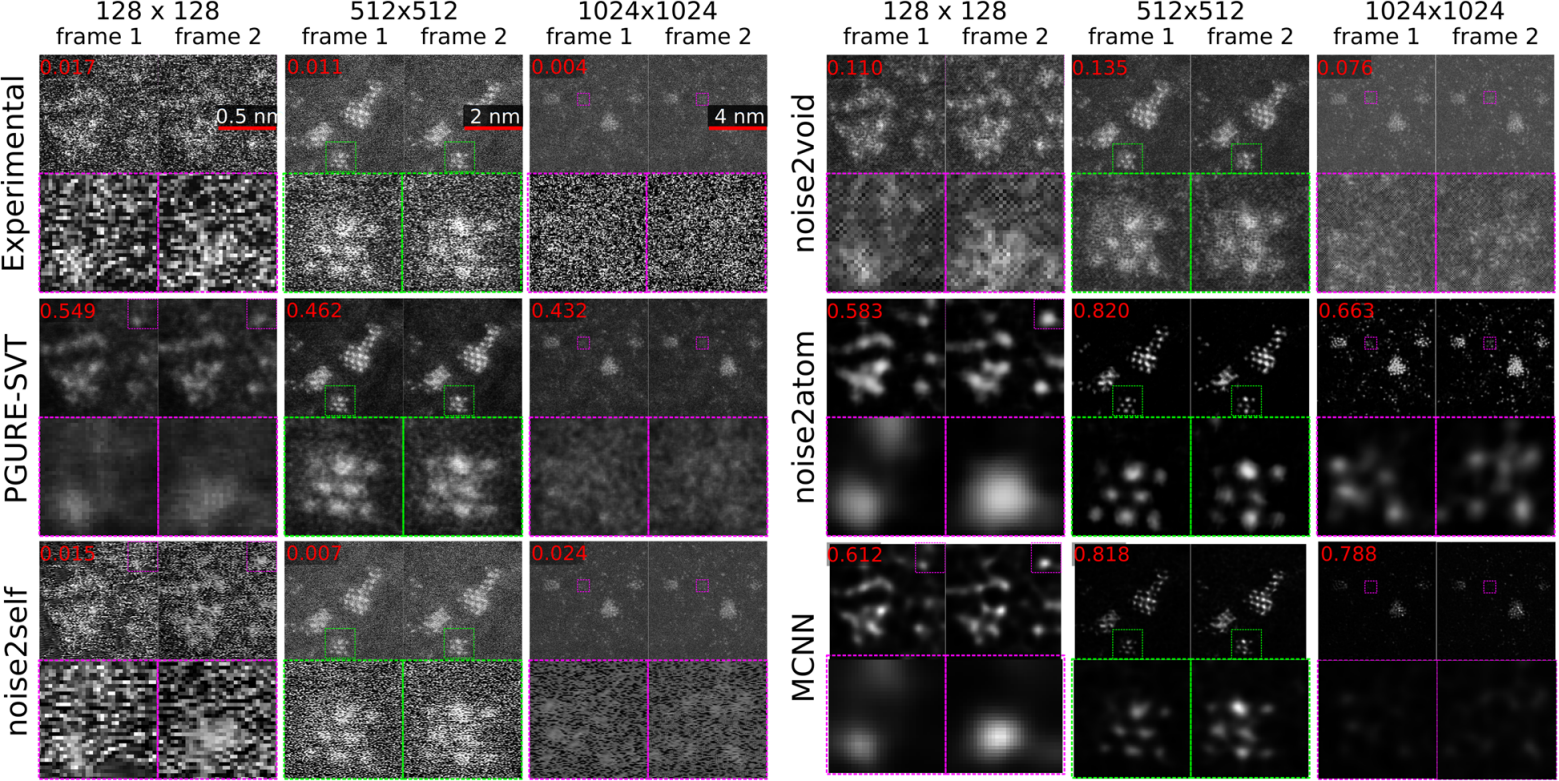
Benchmarking PGURE-SVT, Noise2Self, Noise2Void, Noise2Atom and MCNN on heavily-noised HAADF images. The CSS metrics are presented on the top-left corners. Noise2Atom gives much better predictions than the other unsupervised methods: visually more Gaussian-like and background corrected, with an increased quantitative contrast. In the 512×512 case, Noise2Atom can even beats the baseline MCNN, which has been trained using semi-supervised method

We compared our approach against the recent analytical Poisson-Gaussian Unbiased Risk Estimator for Singular Value Thresholding (PGURE-SVT) method (Furnival et al. 2017) and reasonable deep learning methods Noise2Self (Batson and Royer 2019) and Noise2Void (Krull et al. 2019a). We also tried PPN2V (Prakash et al. 2020) but did not get a satisfying result, as it is challenging to get a good enough parametric noise model estimation. In this benchmark, we used the semi-supervised Multi-scale Convolutional Neural Network (MCNN) method (Wang et al. 2020) as the baseline. When testing with datasets acquired from 150 fps with 128×128 pixels to 5 fps with 1024×1024 pixels, as is shown in Fig. 3, Noise2Atom gives visually clear (Gaussian-like) and consistent (high CSS score) results. And in some case it even outperforms MCNN. Our approach yields predictions reflecting almost only atomic peaks, while removing a vast majority of the background. This result, as our approach consistently detects atoms, agrees well with our physical model. We present more denoising results in noise2atom repository, https://github.com/fengwang/Noise2Atom.

It is increasingly common for a fast modern STEM to produce a dataset including thousands of images in a few minutes. Such a large dataset poses particular computation pressure on Noise2Atom. A direct solution is to sample a fixed number of images, rather than including them all. To find out a lower boundary to make a compromise between the computation speed and denoising quality, we trained four models including 1, 10, 50, and 100 experimental images respectively. Their performances are demonstrated in Fig. 4. From this numeric experiment, we can conclude:

1. A large training set gives good denoising quality. When the training set is small, the denoising quality is not apparently influenced. When equal or less than 50 images are included in the training set, all the CSS metrics are limited to a similar range [0.45,0.48]. But when 100 images are included, the CSS metric reaches 0.69.

**Fig. 4:**
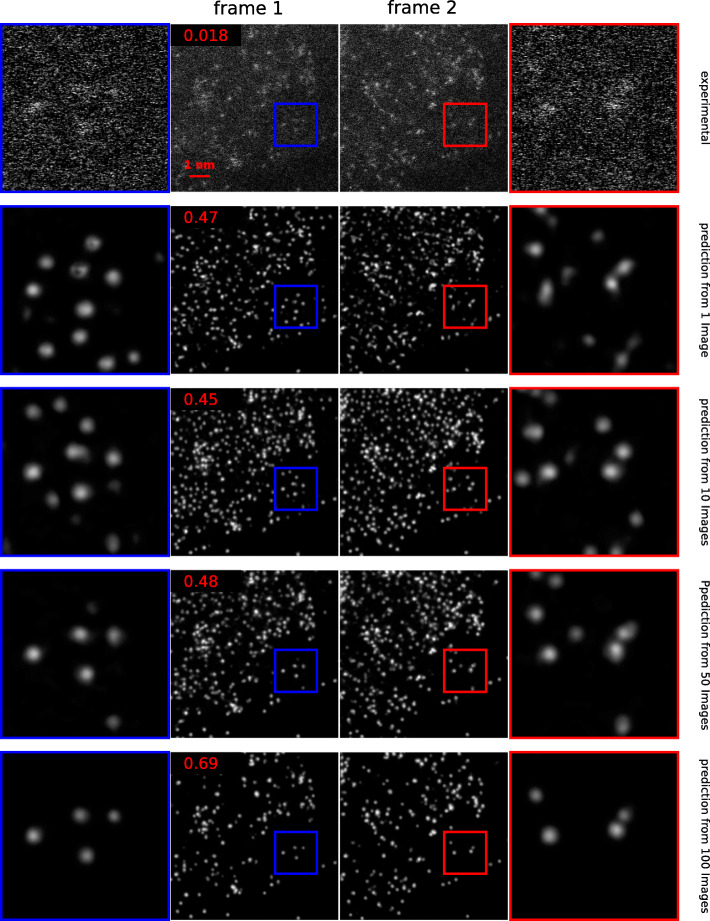
Training Noise2Atom with different numbers of experimental images. The CSS metrics are presented on the top-left corners. The first row presents two consecutive frames of the noisy experimental dataset, with areas of interest amplified. The rest rows present the denoising results from different Noise2Atom models, which are trained using 1, 10, 50 and 100 noisy images respectively
2. A large training set gives robust results: as is demonstrated in the areas of interest in the last row in Fig. 4, the model trained with 100 images predicts four atoms from the first and the second frames. The models trained with small numbers of images tend to over fit atomic peaks onto clusters of bright noisy pixels. As is shown from the second to the fourth row, different numbers of atoms are predicted.

We therefore suggest a training set of around 100 images in favour of both computation speed and denoising quality.

**Fig. 5:**
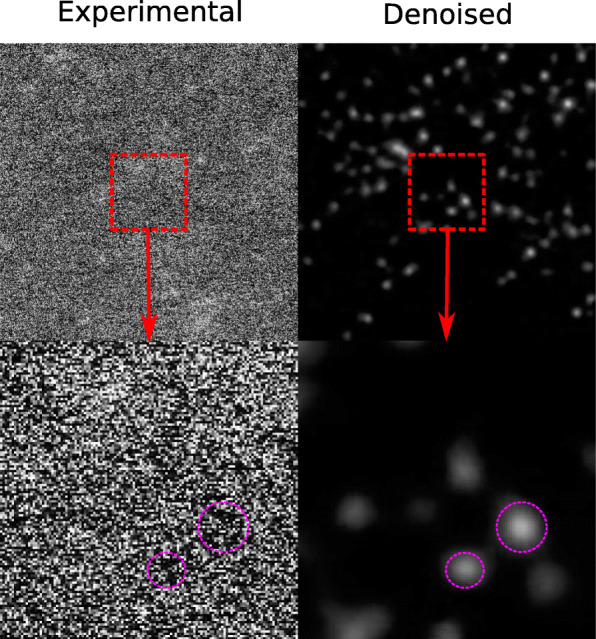
Failure case 1. With too much background noise, bright areas can get mapped to the background, while dark areas get mapped to atoms

**Fig. 6:**
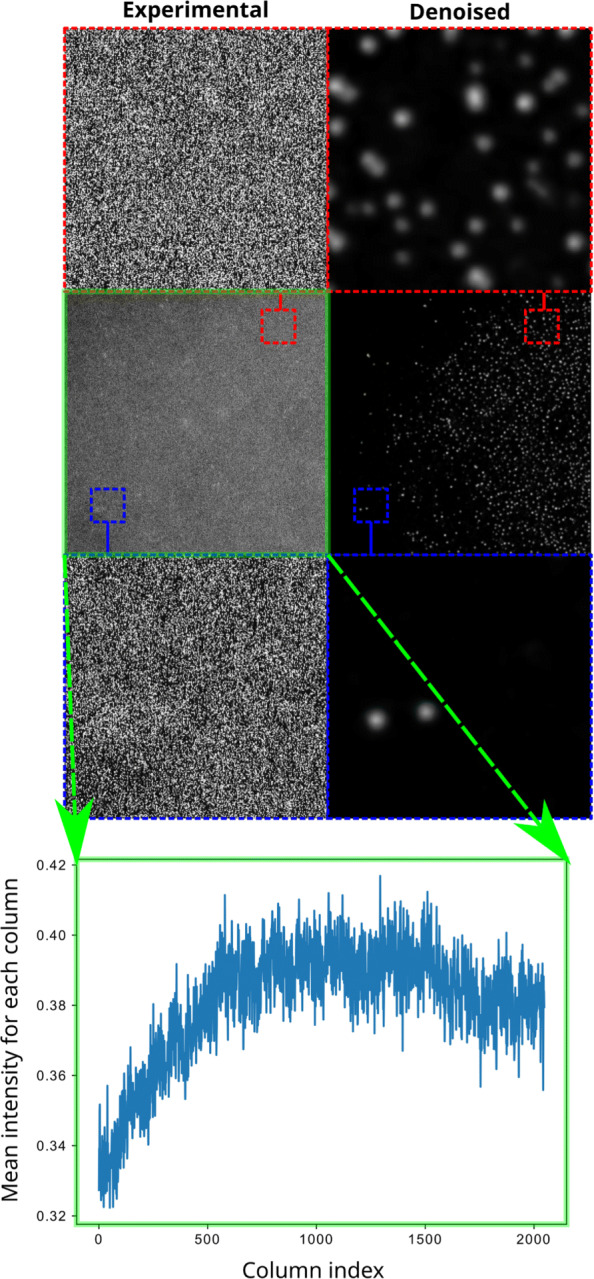
Failure case 2. Noise2Atom is sensitive to gradients in background noise. In this image, at the edge of a nanodroplet with a low concentration of Pt atoms, there is an increase of background noise from left to right (shown in the plot below), as the nanodroplet gets thicker. On the left of the image where a small amount on atoms are correctly fit. However, further to the right of the image, as the background noise increases, clusters of pixels from the background noise, increasingly gets overfit as false atoms

**Failure cases.** As the contrast of STEM depends on atomic number (Kirkland et al. 1987): individual atoms of platinum (Z=78) are reliably detectable with a background signal given by ca 20 nm of carbon (Z=6), as is the case in the results of Fig. 3. This Z-dependence gives an estimated upper detecting limit of maximum ca 40-60 nm of carbon (and other neighboring light elements), before there is too much background noise to reliably detect individual Pt atoms. When we image atoms of Pt in droplets of ionic liquids (up to ca 50nm thickness) on the carbon film (Keller et al. 2019) we get closer to the detection limit, and we can see two failure cases in Figs. 5 and 6. From dark field STEM, we assume that bright areas match atomic peaks, and darker areas are due to the background noise. However, we did not apply an additional constraint to reflect this relationship. In our numerical experiments, occasionally (once in every ten trials), there were cases of reverted mapping: the bright areas go to the background, and the dark areas go to the atomic peaks, as is shown in Fig. 5. A second failure case is due to gradients in the background noise, as shown in Fig. 6. Such a gradient causes areas with higher background noise to be overfit resulting in many false atoms. Therefore, it is important to largely have a homogeneous background intensity across the image.

## Discussion and conclusion

The analytic methods stem from domain knowledge but are computationally inefficient. The supervised learning methods are fast but require paired training sets. Our approach takes advantage of the domain knowledge and is not restricted by the absence of the paired training data. We understand the critic model and the low-frequency feature extraction model as domain knowledge embedding. The critic model gives high priority of 2D Gaussian peaks, which is the expected physical pattern. The low-frequency feature extraction model focuses on the slowly varying features by cutting down the influence of the noises, as the noises take the form of very bright or dark pixels. Our approach shows that even when it is difficult to generate suitable simulated ground truth/noisy image pairs and estimate a proper noise model, it is still possible to train neural networks to restore noisy images to extract interpretable and quantitative information, by using domain knowledge. Hence, the denoising approach of Noise2Atom is especially useful for time-resolved microscopy, but is likely also useful for many other applications. We also make the dataset and source code publicly available.

## Data Availability

The source code and the datasets generated and analysed during the current study are available in the noise2atom repository, https://github.com/fengwang/Noise2Atom.

## Abbreviations

STEM: Scanning transmission electron microscopy
SSIM: Structural similarity index measure
CSS: Consecutive structural similarity
DNN: Deep convolutional neural network
PPN2V: Parametric probabilistic Noise2Void
fps: Frames per second
GAN: Generative adversarial network
PGURESVT: Poisson-Gaussian unbiased risk estimator for singular value thresholding
MCNN: Multi-scale convolutional neural network
HAADF: High-angle annular dark-field

## References

[CR1] M. Arjovsky, S. Chintala, L. Bottou, Wasserstein GAN (2017). arXiv:1701.07875.

[CR2] J. Batson, L. Royer, Noise2Self: Blind Denoising by Self-Supervision (2019). arXiv:1901.11365.

[CR3] T. -O Buchholz, M Jordan, G Pigino, F Jug, in *2019 IEEE 16th International Symposium on Biomedical Imaging (ISBI 2019)*. Cryo-CARE: Content-Aware Image Restoration for Cryo-Transmission Electron Microscopy Data (IEEEVenice, 2019a), pp. 502–506. ISBN 978-1-5386-3641-1. 10.1109/ISBI.2019.8759519.

[CR4] T. -O Buchholz, A Krull, R Shahidi, G Pigino, G Jékely, F Jug, Content-aware image restoration for electron microscopy. Methods Cell Biol.**152:**, 277–289 (2019b). ISSN 0091-679X. 10.1016/bs.mcb.2019.05.001. 10.1016/bs.mcb.2019.05.001 31326025

[CR5] Cao K., Zoberbier T., Biskupek J., Botos A., McSweeney R. L., Kurtoglu A., Stoppiello C. T., Markevich A. V., Besley E., Chamberlain T. W. (2018). Comparison of atomic scale dynamics for the middle and late transition metal nanocatalysts. Nat. Commun..

[CR6] Chang Y, Yan L, Fang H, Zhong S, Liao W (2019). HSI-DeNet: Hyperspectral Image Restoration via Convolutional Neural Network. IEEE Trans. Geosci. Remote Sens..

[CR7] Chollet F (2017). Xception: Deep Learning with Depthwise Separable Convolutions. 2017 IEEE Conference on Computer Vision and Pattern Recognition (CVPR).

[CR8] Dwyer C, Erni R, Etheridge J (2010). Measurement of effective source distribution and its importance for quantitative interpretation of STEM images. Ultramicroscopy.

[CR9] Furnival T, Leary R. K, Midgley P. A (2017). Denoising time-resolved microscopy image sequences with singular value thresholding. Ultramicroscopy.

[CR10] Guo Z., Sun Y., Jian M., Zhang X. (2018). Deep Residual Network with Sparse Feedback for Image Restoration. Appl. Sci..

[CR11] K. He, X. Zhang, S. Ren, J. Sun, in *Proceedings of the IEEE conference on computer vision and pattern recognition*. Deep residual learning for image recognition, (2016), pp. 770–778. 10.1109/cvpr.2016.90.

[CR12] T. R. Henninen, M. Bon, F. Wang, D. Passerone, R. Erni, in *Angewandte Chemie International Edition*. The Structure of Sub-nm Platinum Clusters at Elevated Temperatures, (2019). ISSN 14337851. 10.1002/anie.201911068. 10.1002/anie.201911068 31682061

[CR13] T. R Henninen, D Keller, R Erni, Structure matters – Direct in-situ observation of cluster nucleation at atomic scale in a liquid phase. In review (2020).

[CR14] Kadimesetty V. S., Gutta S., Ganapathy S., Yalavarthy P. K. (2018). Convolutional neural network-based robust denoising of low-dose computed tomography perfusion maps. IEEE Trans. Radiat. Plasma Med. Sci..

[CR15] Keller D., Henninen T. R., Erni R. (2019). Formation of gold nanoparticles in a free-standing ionic liquid triggered by heat and electron irradiation. Micron.

[CR16] Kim T., Cha M., Kim H., Lee J. K., Kim J., Precup D., Teh Y. W. (2017). Learning to Discover Cross-Domain Relations with Generative Adversarial Networks. Proceedings of the 34th International Conference on Machine Learning, vol. 70.

[CR17] Kirkland E. J., Loane R. F., Silcox J. (1987). Simulation of annular dark field STEM images using a modified multislice method. Ultramicroscopy.

[CR18] Kokkinos F, Lefkimmiatis S (2019). Iterative Joint Image Demosaicking and Denoising Using a Residual Denoising Network. IEEE Trans. Image Process..

[CR19] A. Krizhevsky, I. Sutskever, G. E. Hinton, in *Advances in Neural Information Processing Systems, vol. 25*, ed. by F. Pereira, C. J. C. Burges, L. Bottou, and K. Q. Weinberger. ImageNet Classification with Deep Convolutional Neural Networks (Curran Associates, Inc., 2012), pp. 1097–1105. http://papers.nips.cc/paper/4824-imagenet-classification-with-deep-convolutional-neural-networ. Accessed 24 Sept 2020.

[CR20] A. Krull, T. -O. Buchholz, F. Jug, in *The IEEE Conference on Computer Vision and Pattern Recognition (CVPR)*. Noise2void - learning denoising from single noisy images, (2019a). 10.1109/cvpr.2019.00223.

[CR21] A. Krull, T. Vicar, F. Jug, Probabilistic Noise2Void: Unsupervised Content-Aware Denoising (2019b). arXiv:1906.00651.

[CR22] J. Lehtinen, J. Munkberg, J. Hasselgren, S. Laine, T. Karras, M. Aittala, T. Aila, Noise2noise: Learning image restoration without clean data (2018). arXiv preprint arXiv:1803.04189.

[CR23] K. Lin, T. H. Li, S. Liu, G. Li, in *The IEEE Conference on Computer Vision and Pattern Recognition (CVPR) Workshops*. Real photographs denoising with noise domain adaptation and attentive generative adversarial network, (2019). 10.1109/cvprw.2019.00221.

[CR24] P. Liu, H. Zhang, K. Zhang, L. Lin, W. Zuo, in *Proceedings of the IEEE Conference on Computer Vision and Pattern Recognition Workshops*. Multi-level wavelet-CNN for image restoration, (2018), pp. 773–782. 10.1109/cvprw.2018.00121.

[CR25] Lucas A., Iliadis M., Molina R., Katsaggelos A. K. (2018). Using deep neural networks for inverse problems in imaging: Beyond analytical methods. IEEE Signal Process. Mag..

[CR26] B. Mildenhall, J. T. Barron, J. Chen, D. Sharlet, R. Ng, R. Carroll, in *Proceedings of the IEEE Conference on Computer Vision and Pattern Recognition*. Burst denoising with kernel prediction networks, (2018), pp. 2502–2510. 10.1109/cvpr.2018.00265.

[CR27] N. Moran, D. Schmidt, Y. Zhong, P. Coady, in *Proceedings of the IEEE/CVF Conference on Computer Vision and Pattern Recognition*. Noisier2Noise: Learning to Denoise from Unpaired Noisy Data, (2020), pp. 12064–12072. 10.1109/cvpr42600.2020.01208.

[CR28] M Prakash, M Lalit, P Tomancak, A Krul, F Jug, in *2020 IEEE 17th International Symposium on Biomedical Imaging (ISBI)*. Fully Unsupervised Probabilistic Noise2Void, (2020), pp. 154–158. 10.1109/ISBI45749.2020.9098612.

[CR29] Ran M., Hu J., Chen Y., Chen H., Sun H., Zhou J., Zhang Y. (2019). Denoising of 3D magnetic resonance images using a residual encoder–decoder Wasserstein generative adversarial network. Med. Image Anal..

[CR30] O Ronneberger, P Fischer, T Brox, in *Medical Image Computing and Computer-Assisted Intervention – MICCAI 2015, Lecture Notes in Computer Science*, ed. by N. Navab, J. Hornegger, W. M. Wells, and A. F. Frangi. U-Net: Convolutional Networks for Biomedical Image Segmentation (Cham. Springer International Publishing, 2015), pp. 234–241. ISBN 978-3-319-24574-4. 10.1007/978-3-319-24574-4_28.

[CR31] Song Y, Zhu Y, Du X (2019). Dynamic Residual Dense Network for Image Denoising. Sensors.

[CR32] Su Y., Lian Q., Zhang X., Shi B., Fan X. (2019). Multi-scale cross-path concatenation residual network for Poisson denoising. IET Image Process..

[CR33] D. Ulyanov, A. Vedaldi, V. Lempitsky, *Instance normalization: The missing ingredient for fast stylization*, (2016). arXiv preprint arXiv:1607.08022.

[CR34] Wang F, Eljarrat A, Müller J, Henninen T. R, Erni R, Koch C. T (2020). Multi-resolution convolutional neural networks for inverse problems. Sci. Rep..

[CR35] J. Wu, Z. Huang, J. Thoma, D. Acharya, L. Van Gool, in *Proceedings of the European Conference on Computer Vision (ECCV)*. Wasserstein divergence for gans, (2018), pp. 653–668. 10.1007/978-3-030-01228-1_40.

[CR36] Xie W., Li Y., Jia X. (2018). Deep convolutional networks with residual learning for accurate spectral-spatial denoising. Neurocomputing.

[CR37] Z. Yi, H. Zhang, P. Tan, M. Gong, in *Proceedings of the IEEE International Conference on Computer Vision*. Dualgan: Unsupervised dual learning for image-to-image translation, (2017), pp. 2849–2857. 10.1109/iccv.2017.310.

[CR38] Zhang K, Zuo W, Zhang L (2018). FFDNet: Toward a Fast and Flexible Solution for CNN based Image Denoising. IEEE Trans. Image Process..

[CR39] Y. Zhang, Y. Zhu, E. Nichols, Q. Wang, S. Zhang, C. Smith, S. Howard, in *Proceedings of the IEEE Conference on Computer Vision and Pattern Recognition*. A poisson-gaussian denoising dataset with real fluorescence microscopy images, (2019), pp. 11710–11718. 10.1109/cvpr.2019.01198.

[CR40] J. -Y. Zhu, T. Park, P. Isola, A. A. Efros, in *Proceedings of the IEEE International Conference on Computer Vision*. Unpaired image-to-image translation using cycle-consistent adversarial networks, (2017), pp. 2223–2232. 10.1109/iccv.2017.244.

